# Recent insights in the role of biomarkers in severe asthma management

**DOI:** 10.3389/fmed.2022.992565

**Published:** 2022-09-26

**Authors:** Evangelia Fouka, Kalliopi Domvri, Foteini Gkakou, Maria Alevizaki, Paschalis Steiropoulos, Despoina Papakosta, Konstantinos Porpodis

**Affiliations:** ^1^G. Papanikolaou General Hospital, Thessaloniki, Greece; ^2^Pulmonary Department of Aristotle University of Thessaloniki, Thessaloniki, Greece; ^3^Department of Medicine, Democritus University of Thrace, Alexandroupolis, Greece

**Keywords:** biomarkers, severe asthma, T2 asthma, non-T2 airway inflammation, omics, microbiome & dysbiosis

## Abstract

Contemporary asthma management requires a proactive and individualized approach, combining precision diagnosis and personalized treatment. The introduction of biologic therapies for severe asthma to everyday clinical practice, increases the need for specific patient selection, prediction of outcomes and monitoring of these costly and long-lasting therapies. Several biomarkers have been used in asthma in disease identification, prediction of asthma severity and prognosis, and response to treatment. Novel advances in the area of personalized medicine regarding disease phenotyping and endotyping, encompass the development and application of reliable biomarkers, accurately quantified using robust and reproducible methods. The availability of powerful omics technologies, together with integrated and network-based genome data analysis, and microbiota changes quantified in serum, body fluids and exhaled air, will lead to a better classification of distinct phenotypes or endotypes. Herein, in this review we discuss on currently used and novel biomarkers for the diagnosis and treatment of asthma.

## Introduction

In the past decades, asthma has been increasingly recognized as a heterogeneous disease, with many diverse pathogenetic mechanisms and variable responses to standard therapy (1). Targeting of the underlying inflammatory pathways is the current therapeutic approach in asthma, especially for patients with severe or difficult-to-control disease (2).

Particularly in severe asthma, diverse inflammatory pathways may be activated in different patient subsets, leading to the emergence of distinguished clinical characteristics or phenotypes (3). Based on cluster analysis of several large asthmatic cohorts, we have identified four major clinical phenotypes in adult patients with severe asthma, considering relevant observable characteristics such as age at onset, potential triggers, type of inflammation, lung function impairment, and response to treatment: (a) the early-onset, atopic asthma phenotype, presenting partially variable airflow obstruction, frequent symptoms and relative response to corticosteroid treatment, (b) the late-onset, non-atopic, eosinophilic phenotype, with fixed airway obstruction, corticosteroid resistance and frequent asthma-related comorbidities (c) the late-onset, non-allergic, non-eosinophilic, obesity-related asthma phenotype, usually female predominant, with increased symptom burden and resistance to corticosteroids despite relatively normal lung function, and (d) the late-onset, non-atopic, neutrophilic phenotype, with corticosteroid resistance and severe lung function impairment (4–6). However, considerable overlapping between asthma phenotypes is often observed, and this phenomenon may be attributed to the different variables assessed in various studies and ethnic, geographical and other methodological issues.

Moreover, based on the presence or absence of Type 2 (T2) inflammation, asthma is now frequently categorized into T2 high (T2 asthma) and T2 low (non-T2 asthma) (7). The characterization of the distinct disease endotypes, that is the underlying pathophysiologic mechanisms, has currently become the central therapeutic strategy in asthma management, as it enables clinicians to better diagnosing, stratifying and monitoring of their patients (8).

Consequently, the clinical and pathophysiological heterogeneity of asthma makes it extremely suitable for precision medicine (9). Biological markers (biomarkers), defined as measurable indicators of a biological state or disease with clear cutoff values (10), are considered valuable clinical tools for diagnosing, predicting, and monitoring asthma, with the aim to reduce its burden and to minimize future risk (11).

**Figure 1:**
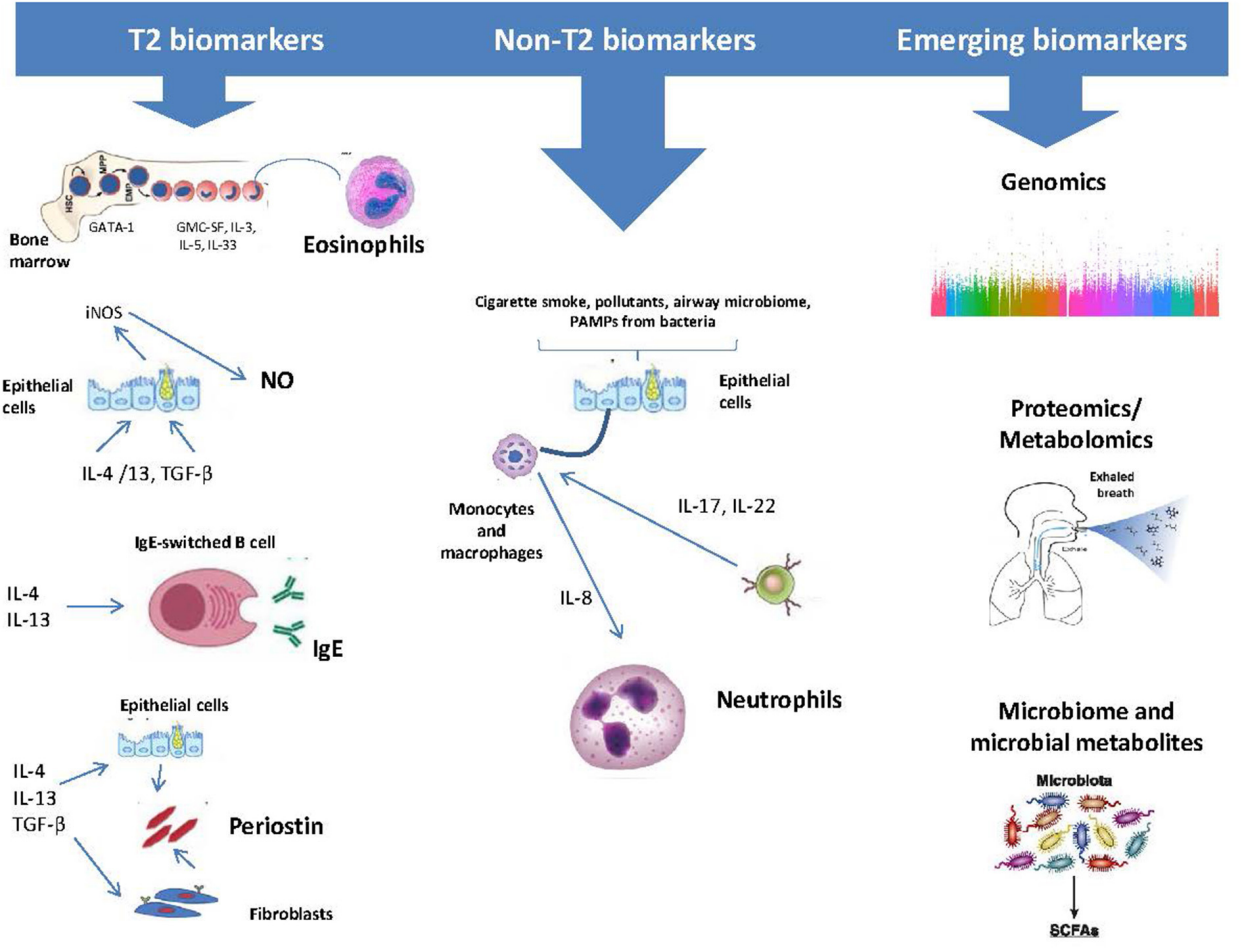
Asthma biomarkers categorized as those related to type 2 (T2) Inflammation and those that relate to other biological processes.

During the last decades, as the contribution of atopy, eosinophilic-driven inflammation and airway epithelial dysfunction have been recognized in the pathogenesis of severe asthma, significant progress has been made in the identification of valid asthma biomarkers (12, 13). Hence, serum immunoglobulin E (IgE), sputum and blood eosinophils, and the fraction of nitric oxide in exhaled air (FeNO), have all been used as potential biomarkers, suggestive of the underlying activation of these respective pathogenetic pathways (14–17). In this review, we aimed to discuss on the most important existing and emerging biomarkers with the greater clinical applicability in asthma (Figure 1).

## Biomarkers in T2 asthma

At present, T2 asthma comprises the best defined asthma subtype, regarding underlying immunopathology, associated biomarkers and targeted therapies (18). Airway eosinophilic inflammation constitutes the main characteristic of this type of asthma, so the latter is often classified merely as eosinophilic or non-eosinophilic (19). Eosinophilic inflammation is driven predominantly by T2 immune responses regulated by T2-helper (Th2) cells and group 2 innate lymphoid cells (ILC-2) and their relative Th2 cytokines, and it is reflected, to some extent, by sputum or blood eosinophilia and elevated FeNO concentration (20).

### Eosinophils

Among the asthmatic patients, the prevalence of eosinophilic inflammation is referred as almost as 50% in various studies (21). Upon their activation, eosinophils release a number of inflammatory mediators from their intracellular granules, that are rsponsible not only for the appearance of asthmatic symptoms, but also, in more advanced stages of the disease, for the observed fixed airflow obstruction and airway remodeling (22). IL-5, IL-4, and IL-13 are key cytokines in the eosinophilic inflammatory process, as they regulate the eosinophilic migration, activation and survival into the asthmatic airway (23).

The association between asthma and eosinophilia is well-established. Airway eosinophilia is significantly increased in asthmatic patients compared with healthy subjects, and has been found to correlate with the degree of airway hyperreactivity (AHR), exacerbation severity and poor clinical outcomes (24, 25). Sputum eosinophilia as an index of airway eosinophilia, has been found to predict response to treatment with corticosteroids (26) and may be used as a therapeutic target for guiding the intensity of treatment with inhaled corticosteroids (ICS) (27). Additionally, treatment adjustments according to sputum eosinophil levels have been associated with reductions in the rate of severe exacerbations and with decreased need for hospital admissions, compared to the standard clinical-guided management, in patients with moderate-to-severe asthma (28).

Pizzichini et al., in a small cross-sectional study suggested that sputum eosinophilia (cut-off >3%) is a more reflective biomarker of asthmatic airway inflammation than the absolute peripheral blood eosinophil count (BEC) (29). However, sputum eosinophil levels have been found to vary over time and after treatment (30), and induced sputum technique may also be laborious and time-consuming. Moreover, a recent *post-hoc* analysis of data from patients who had provided both baseline sputum and blood samples participating in DREAM study (31) revealed that, among the placebo group, increasing baseline sputum eosinophil count predicted a decrease in exacerbation rate in the following 52 weeks, while, on the contrary, predicted exacerbations increased with increasing baseline BEC (32). The authors of the study suggest that this finding may be due to the small number of patients included in the analysis; however, this may also represent an interesting new finding suggesting that sputum eosinophils may not represent a more useful predictive biomarker than BEC in patients with severe eosinophilic asthma. Furthermore, BEC is considered superior to FeNO in identifying sputum eosinophilia (33) and, most importantly, is easily measured and widely used in the context of whole blood count testing; therefore a recent (or historical) BEC is usually available for most the patients. For these reasons, the use of BEC as the basic biomarkers of T2 inflammation has now prevailed in daily clinical practice.

From a clinical standpoint, BEC are useful in the early detection of exacerbation risk, loss of asthma control and lung function deterioration in patients with severe asthma (34, 35). Baseline BEC have been shown to predict the degree of reduction in severe exacerbation rate in patients with severe eosinophilic asthma treated with ICS (36). BEC values between 150 and 400 cells/μL have also been used to predict response to treatment with monoclonal antibodies (mAbs) targeting IL-5 and its receptor IL-5R (16, 37–39). In the pooled analysis of the DREAM and MENSA studies (37), a better response to treatment with the anti-IL5 mAb mepolizumab was observed in patients with severe asthma and BEC of ≥150 cells/μL in baseline. Similarly, data from the pooled analysis of the CALIMA and SIROCCO studies showed that BEC ≥ 300 cells/μL, along with the rate of exacerbations during the previous year, were the stronger predictors of treatment response to benralizumab, an anti-IL5R biologic (16). In the early efficacy studies of the anti-IL5 mAb reslizumab, significant reductions in asthma exacerbation rate (38) and improvements in lung function (39) were observed in patients with persistent asthma and BEC ≥400 cells/μL. However, BEC should be considered a continuous variable, with higher levels predicting a greater response to treatment (40).

The inconsistency observed between sputum and blood eosinophil counts may be attributed to the heterogeneity of the studied populations in several studies (41). Moreover, a single BEC measurement may be insufficient for asthma diagnosis and therapeutic decisions, with greater variability observed for BEC values between 150 and 299 cells/μL (42). Finally, the utility of BEC also seems to be restricted by their considerably low specificity, as elevated levels can be observed in several autoimmune and atopic diseases and in many parasitic infections (43).

### FeNO

In asthma, the bronchial airway is usually rich in Nitric oxide (NO), produced from inducible nitric oxide synthases (iNOS) under the mediation of the T2 inflammatory cytokines IL-4 and IL-13 (44). NO has been used as an indirect marker of asthmatic inflammation, as its fraction in exhaled breath (FeNO) can be measured non-invasively with the use of a hand-held analyzer, making it a valuable clinical tool in everyday clinical practice (45).

However, FeNO can only modestly predict airway eosinophilia, with many studies suggesting moderate sensitivity and specificity for detecting sputum eosinophilia ≥3% (46). Several cut-off values have been proposed, though it has become evident that values < 25 ppb generally rule out airway eosinophilic inflammation, whereas values >50 ppb are strongly suggestive (47).

Elevated FeNO levels may reflect ongoing airway inflammation and may predict an increased risk for asthma exacerbations and decline in lung function (48, 49). In steroid-naive asthma patients, FeNO levels are higher in patients with allergic asthma and are associated with greater AHR and reversibility of airway obstruction (50). A FeNO level >50 ppb has been found predictive of response to ICS (47) and, in the appropriate clinical setting, may support the decision for the initiation of ICS. FeNO levels are usually responsive to ICS therapy and their changes have been used to monitor adherence to treatment in patients with uncontrolled asthma (51). In an 8-week, randomized, placebo-controlled trial, FeNO levels significantly decreased and then remained stable after only 2 weeks of treatment with ICS compared with placebo, while they again significantly increased shortly after the 2-week washout period (52). In an Australian RCT including 220 pregnant, non-smoking asthmatic women, 53% of whom had non-eosinophilic, treatment adjustments made according to FeNO levels resulted in more significant reductions in exacerbation rates compared to clinical assessment only guided-therapy (53). A 36-week double-blind, parallel RCT comparing FeNO-guided vs. symptom-based management in 80 asthmatic adults with a history of atopy in two-thirds (54), demonstrated a significant decrease in AHR at the end of the study in the FeNO group compared with the control group. In a more recent double-blind, multicenter RCT, baseline FeNO levels were useful in predicting response to treatment with ICS in patients with no previous asthma diagnosis, non-specific respiratory symptoms (cough, wheeze, or dyspnea), and < 20% bronchodilator reversibility, mean BEC < 260 cells/μL, after exclusion of COPD diagnosis (55). The findings of these studies indicate that the use of FeNO in guiding asthma management may be more appropriate in atopic patients.

However, although airway eosinophilia and T2-inflammation may overlap, they are not synonymous. That has been clearly illustrated in the early mepolizumab (anti-IL5 mAb) and Dupilumab (anti-IL4Rα mAb) studies, in which blood eosinophil and FeNO levels, respectively, were affected by therapy to a different extent (15, 17). In addition, the inconsistency between FeNO levels and asthma control may be, at least partially, explained by the fact that numerous factors, such as age, gender, body weight, atopy, smoking, food and upper respiratory allergic diseases, can affect FeNO and should be taken into account in clinical decisions (56). Therefore, the 2021 National Asthma Education and Prevention Coordinating Committee (NAEPPCC) made a conditional recommendation for FeNO use in clinical practice, suggesting its use in combination with compatible clinical parameters including history, symptoms and clinical and spirometric findings (46).

### IgE

The role of IgE in atopic asthma has been well-described for many decades (57). However, although robust evidence of the observed immunological changes produced by anti-IgE therapies have been produced by the times of the first clinical studies of omalizumab (14, 58), the utility of IgE as a biomarker of allergic inflammation in asthma has not yet been fully clarified. However, it has been suggested that total IgE levels can serve as a biomarker for monitoring IgE production and treatment guidance in an individual level (59).

In allergic asthmatics, several studies have shown an association between allergic sensitization in early life and lung function impairment in adulthood”. (60–63). Total serum IgE levels have been shown to correlate with asthma severity, and with increased risk for loss of asthma control and severe exacerbations both in children and adults (64–66). However, an old study by the TENOR study group, did not demonstrate a relationship between total IgE geometric mean and severity of asthma, although IgE total levels were higher in children with severe asthma compared to those with mild-moderate disease (67). Moreover, therapeutic efficacy of omalizumab has been shown to be comparable in patients with baseline IgE levels between 30 and 700 UI/mL (68, 69), therefore IgE cannot reliably be used as a biomarker for prediction of treatment response in omalizumab.

On the contrary, measurement of specific IgE (sIgE) to aeroallergens has shown relatively high sensitivity concerning asthma diagnosis (70). Furthermore, Matsui et al. (71), demonstrated that sIgE levels were associated with more severe asthma in a large cohort of young inner-city asthmatics. Similarly, in a more recent study, the number of specific allergen sensitizations correlated with asthma severity and exacerbation risk in children (72). As total serum IgE is not allergen-specific and may be influenced by several extrinsic factors and pathologic conditions, sIgE may be considered a more reliable biomarker both for asthma diagnosis and severity assessment.

### Periostin

Periostin is a secreted extracellular matrix protein produced by bronchial epithelial cells and fibroblasts under the stimulus of IL-4 and IL-13, therefore it is also considered to be a T2-related biomarker (73). However, periostin differentiates from other T2 biomarkers in that it is implicated in airway remodeling, therefore it is considered to be associated with chronic rather than acute T2 inflammation (74).

Serum periostin has been associated with fixed airflow limitation and subepithelial fibrosis in a number of clinical studies (75, 76). An RCT evaluating the effect of ICS therapy on serum periostin levels revealed that, ICS significantly lowered both serum periostin and sputum eosinophil counts, and that decrease was associated with improvements in lung function and airway remodeling (77).

However, several studies underline the existing discordance between serum periostin and sputum eosinophilia. Findings from the BOBCAT study showed that periostin was a good predictor of airway eosinophilic inflammation, with an AUROC of 0.84 (78), however, in another study, periostin was unable to differentiate eosinophilic from non-eosinophilic asthma (33). Moreover, wide periostin use is confined by the lack of standardized measurement techniques and validated predicted values (79).

Anti-IL-13 therapies have not so far been authorized for the treatment of severe eosinophilic asthma. However, in a phase-2 RCT including 219 patients with uncontrolled asthma, lebrikizumab, an anti-IL13 mAb, significantly increased FEV1 compared with placebo, only in patients with high serum periostin levels at baseline (80). In a 52-week RCT of tralokinumab, another anti-IL13 mAb, significant improvements in asthma exacerbation rate, lung function, and asthma symptoms were also found in patients with increased pre-treatment periostin serum levels (81). Therefore, periostin is also an emerging biomarker of T2 inflammation.

A summary of the main characteristics of the T2-biomarkers presented above is illustrated in Table 1.

**Table 1:** Summary of characteristics of major T2-biomarkers.

**Biomarker**	**Utility**	**Advantages**	**Limits**
Sputum eosinophils counts	Define the airway eosinophilic phenotype (29). Predict AHR, severity of exacerbations and disease outcomes (24, 25). Predict response to treatment with CS (26).	Non-invasive	Time consuming, requires specialized equipment, not all patients can provide adequate samples. Time and treatment-dependent variations (30)
Blood eosinophils counts	Define the eosinophilic phenotype (33). Predictive of exacerbations, loss of asthma control and lung function decline (32, 34, 35). Predict response to biologic therapies (16, 36–40)	Minimally invasive Easily measured in the clinical setting. Good correlations with sputum eosinophils compared to FeNO (33)	Varying cut-off points, variable stability over time (42), relatively low specificity (43)
FeNO	Identifies airway eosinophilic inflammation (45). Prognostic of exacerbations, lung function decline, and degree of AHR (48–50). Predicts response to treatment with CS and biologics (17, 47, 55) and detects adherence to treatment (51) and treatment success (5–54).	Non-invasive. Easily collected in clinical setting	Moderate sensitivity and specificity for sputum eosinophilia ≥3% (46). Affected by multiple confounders (56)
Total IgE	Predictive of asthma severity and risk for exacerbations and loss of asthma control (64–66).	Minimally invasive. Identifies patients eligible for anti-IgE therapy (14, 58)	Not-predictive of response to anti-IgE therapy (68, 69). Not asthma-specific; outperformed by sIgE in diagnosing asthma and predicting asthma severity and exacerbation risk (70–72)
Periostin	Identifies T2-inflammation (73), airway remodeling and fixed airway obstruction (74–76). Predicts response to treatment with anti-IL-13 biologicals (80, 81).	Not currently available in clinical practice. Lack of standardized measurement techniques and reference values (79)	Poorly associated with sputum eosinophilia (33, 78)

FeNO, fraction of nitric oxide in exhaled air; IgE, immunoglobulin E; sIgE, specific immunoglobulin E; CS, corticosteroids; AHR, airway hyperreactivity.

## Biomarkers in non-T2 asthma

Non-eosinophilic asthma has traditionally been defined as asthma without evidence of T2 inflammation, and in general is characterized by the predominance of neutrophils or the absence (or normal levels) of other granulocytes in induced sputum (82).

Neutrophilic phenotype constitutes a proportion of non-T2 asthma (83), although until recently it was thought to be a misdiagnosis of chronic obstructive pulmonary disease (COPD) or a consequence of corticosteroid treatment (84). The normal range of neutrophils in induced sputum in healthy individuals ranges between 30 and 50%, and subsequently airway neutrophilia is defined as between 51 and 70% (85). However, age-specific reference values are warranted, as airway neutrophilia varies with age (86). The neutrophilic phenotype is widely associated with smoking, obesity, acute airway infections, underlying anti-inflammatory therapies and various forms of air pollution (87, 88). Airway neutrophilia has been found to be facilitated by local, IL-17 mediated (89) and systemic inflammatory pathways (90).

Paucigranulocytic asthma comprises another component of non-T2 asthma, and is by default defined as asthma without T2 biomarkers (91). Therefore, targeted treatment options, as well as clinically applicable biomarkers, are still largely under exploration (92). In this context, the clinically relevant issue of whether “true” non-T2 asthma really exists has been raised, as high-dose ICS and oral corticosteroids (OCS) may potentially minimize blood eosinophils and FeNO levels, therefore masking preexisting T2 inflammation (93).

However, “prototype” non-T2 asthma is associated with poor response to corticosteroid treatment (94), therefore some authors suggest that high doses of ICS may be reduced in the majority of patients with non-eosinophilic asthma (95), and in the absence of targeted therapies, other treatable traits should be investigated e.g., exposure to smoking, obesity, chronic respiratory infections, and airway smooth muscle dysfunction (96). Although smoking cessation is favorable for all asthmatic patients, independently of their inflammatory phenotype, this intervention might be even more important for asthmatic patients with neutrophilic inflammation (97). Similarly, although obesity has been linked with neutrophilic inflammation, there is evidence that weight loss interventions may reduce symptom burden in obese asthmatics through inflammation-independent pathways (98). Long-term, low-dose macrolide therapy may also be a therapeutic option for this subset of patients. In the AMAZES study (99), long-term, low does oral azithromycin therapy significantly decreased exacerbations and improved quality of life in patients with uncontrolled asthma, irrespectively of the underlying inflammatory phenotype. A range of biologics targeting non-T2 cytokines, such as IL-17, IL-6, IL-1, and TNFα, have been tested in several clinical trials, but none of them has shown particular benefits for non-T2 asthma (100).

## Emerging biomarkers

### The omics approach

As our understanding of asthma heterogeneity increases, it has become evident that best clinical practice demands new approaches in the era of personalized medicine. The omics technologies currently emphasize on the identification of clinically applicable proteomic and genomic-based biomarkers to facilitate health-care development (101).

### Genomics

The chromosome 17q21 is in the epicenter of current genomic epidemiological studies in asthma, as it encloses many genes and single nucleotide polymorphisms (SNPs) of interest (102). Several genes (CLC, EMR4P, IL- 5RA, FRRS1, HRH4, SLC29A1, SIGLEC8, and IL1RL1) have been identified to be overexpressed in allergic diseases such as asthma, dermatitis and rhinitis, with IL-5/JAK/STAT and IL-33/ST2/IRAK/TRAF pathways being central in disease multimorbidity, both in children and adoloscents (103). Similarly, genetic variations of the interleukin-1 receptor-like 1 (IL1RL1) gene have also been related with asthma exacerbations in children (104).

However, although transcriptomics studies have been valuable in the characterization of genome variations between the different asthma subtypes, asthma susceptibility cannot be linked to just a number of genetic signatures, due to the complex underlying disease-pathogenetic mechanisms (105). Therefore, genome-wide, large-scale approaches are essential, such as those performed in the U-BIOPRED consortium (106), which evaluated over 1,500 differentially expressed genes from large asthma cohorts and revealed three distinct clusters of disease: (i) an eosinophilic cluster, characterized by the IL-33R, CCR3, and thymic stromal lymphopoietin (TSLP) receptor (TSLPR) transcript expression, that was associated with the highest sputum eosinophilia, more elevated FeNO levels, and more severe asthma; (ii) a neutrophilic cluster, characterized by overexpression of genes related to interferon (IFN) and tumor necrosis factor (TNF), sputum neutrophilia, high levels of systemic inflammation and prevalence of eczema; and (iii) a paucigranulocytic-eosinophilic cluster, characterized by genes regulating various metabolic pathways and mitochondrial functions, that shows the lowest prevalence in severe asthma.

### Proteomic/metabolomics

In recent years, both proteomics and metabolomics technologies have been increasingly used for the recognition of novel biomarkers in asthma. Exhaled breath, mainly comprised of water vapor and inert gases, also contains thousands of volatile organic compounds (VOCs) (107), with pH, H2O2, and several indirect by-products of oxidation, like 8-isoprostane and ethane in exhaled breath, being the most often analyzed by breathomics technology in asthma (108). Exhaled VOC analysis technologies include two main methodological headings approaches: (a) pattern recognition sensors termed “electronic noses” (e-noses), which “signal” the differential of multiple overlapping VOCs, and (b) chemical analytical techniques, typically with the use of mass spectrometry, that measure ions created by VOCs according to their mass/charge ratio (109). Emerging evidence supports the hypothesis that measurement of VOCs concentrations alterations in exhaled air of asthmatic patients may reflect different disease states, suggesting potential usefulness in non-invasive, early diagnosis and effective management (110–112).

Abdel- Aziz et al. (113) used an electronic nose tool to successfully distinguish healthy from asthmatic subjects, while Nieto-Fontarigo et al. (114), combining liquid chromatography with mass spectrometry, identified 18 serum proteins as potential biomarkers of asthma phenotypes (e.g., FCN2 and MASP1 for non-allergic asthma, or HSPG2 and IGFALS for allergic asthma) and disease severity. Moreover, exhaled breath pH has been found significantly lower in severe refractory asthma compared to moderate disease and healthy subjects (115) and in asthmatic smokers compared with non-smokers (116). Lower EBC pH values have been associated with high BMI, high BAL neutrophil counts, impaired lung function, and increased allergic and gastroesophageal reflux symptoms (117). Brinkman et al. have also illustrated differences in exhaled volatiles such as methanol, acetonitrile, and bicyclooctan-1-ol, 4-methyl in patients with loss of asthma control compared with the recovery period (111). On the contrary, the results of the ALLIANCE study failed to show differences in exhaled VOCs and inflammatory markers in asthmatic patients (118). Although further work is required to validate these preliminary findings, several systematic reviews suggest the large potential for the breathomics biomarker approach (112).

### Microbiota and respiratory infections biomarkers

Modern lifestyle has fundamentally disrupted the human microbiome ecology and this may explain, at least in part, the increased incidence of immune-mediated diseases such as allergy and asthma (119). These highly sophisticated host–microbe interactions are currently being intensely studied by many researchers around the world.

It is evident that early-life alterations in gut microbiome composition may be involved in asthma pathogenesis (120). In adult asthmatics, a positive correlation between the increased abundance of histamine-secreting bacteria in the gut and asthma severity has been found (121). Similarly, in murine models of asthma, bacterial-derived histamine released within the gut has been demonstrated to mediate significant inflammatory responses in the lungs (122). In addition to increased abundance, more interestingly, decreased microbiome diversity in the gut has been associated with dysbiosis and enhanced prevalence of allergies and asthma (123, 124). Therefore, due to the close interaction of intestinal microbiota on the mucosal immune system, the gut can be considered as an essential site of immune cross-talk, with an opposing impact on allergy development and treatment, the so-called gut-lung axis (125).

In addition to the gut microbiota, several studies have also showed significant alterations in the microbial communities in the asthmatic airways. Zhou et al., have shown that the relative abundance of *Veillonella* and *Prevotella spp* in the airways of newborns was associated with increased risk of asthma incidence by the age of 6 years (126). In another study, alterations in the microbiome composition in the upper airways in infants were associated with a higher risk of severe asthma exacerbation in asthmatic children (127). In adults, predominance of the phylum *Proteobacteria* in the lungs may be associated with more severe asthma or with loss of asthma control (128), with more profound microbial changes observed in obese asthmatics (129). Microbial metabolites may also potentially serve as valuable biomarkers in asthma, as dysfunctional relationships have been found between respiratory microbes and their circulating metabolites (130).

## Limitations and future perspectives

Unfortunately, at the moment, all the available asthma biomarkers used in severe asthma lack of specificity for the identification of the precise disease endotype that will respond to the existing targeted treatments i.e., blood eosinophilia predicts therapeutic response to all currently available biological therapies (10). Similarly, biomarkers cannot differentiate eosinophilic asthma from eosinophilic COPD, although the relationship between airway and systemic eosinophilia and T2 inflammation appears stronger in severe asthma than in COPD (131). In line with the above, the use of blood eosinophils as the sole biomarker for the eosinophilic phenotype may be misleading, as the amount of eosinophils in the blood is both time and treatment-dependent (132, 133), therefore a single low BEC may not necessarily rule out airway eosinophilia (134).

In recognition of the inherent heterogeneity of asthma phenotypes, Pavord et al. (135), proposed a gradient rather than a dichotomous approach for the classification of severe asthma phenotypes. Similarly, Heaney et al. (136), using data of 1,716 patients from the International Severe Asthma Registry (ISAR), proposed an eosinophilic-probability algorithm to assess severe asthma phenotypes, classifying eosinophilic status from Grade 3 (most likely eosinophilic) to Grade 0 (non-eosinophilic). With the use of this approach, the authors showed that the eosinophilic phenotype prevails in the majority of severe asthmatics, with 83.8% of subjects belonging into the “most likely” eosinophilic phenotype and only 1.6% characterized as “non-eosinophilic”. Supportive evidence on this issue comes from the real-world Wessex AsThma CoHort of difficult asthma (WATCH) study (137), that searched historical electronic blood count records of patients with difficult asthma over a period of 10 years, and reported a strikingly increased prevalence of “eosinophilia ever” when viewed longitudinally.

The combination of different biomarkers may add additional discriminatory value in predicting exacerbations and response to treatment. Price et al., in an observational study with participation of primary care patients, demonstrated that the combination of both high FeNO (>50 ppb) and high BEC (>300 cells/μL) resulted in an almost 4-fold greater exacerbation risk the preceding year, compared to patients with the low biomarkers reference group (138).

More interestingly, the combination of biomarkers with relevant clinical characteristics may be more accurate in the characterization of asthma phenotypes. Recently, a severe asthma expert consensus proposed a set of diagnostic criteria for severe eosinophilic asthma, however, this approach has not been validated in a clinical setting (139). To this point, the ISAR study group (140), developed a multicomponent algorithm for use in real life, incorporating many variables associated with severe eosinophilic asthma (BEC FeNO, atopic status, age of asthma onset, asthma related comorbidities, and OCS use), while both the Severe Asthma Research Program (SARP) and the Leicester cohorts have also used this approach (141). Based on the combination of feasible biomarkers with clinical meaningful disease characteristics, current asthma guidelines have now adapted algorithms for the initial choice of targeted biologic treatments and for the monitoring of subsequent treatment response (142–144).

## Conclusions

Biomarkers are significant elements of precision medicine, as they may provide valuable information, in terms of characterization of disease phenotypes and endotypes, selection of the appropriate targeted therapy, and monitoring of treatment efficacy. The advent of new techniques, combined with biomarker combination strategies, will likely assist the identification of novel functional traits for patients non-responsive to currently available targeted treatment options, including patients with non-T2 asthma.

## Author contributions

EF contributed to conception of the manuscript and wrote the first draft of the manuscript. EF, FG, and MA wrote sections of the manuscript. KD and KP have also contributed to conception of the manuscript, apart from revision, read and approve. All authors contributed to manuscript revision, read, and approved the submitted version.

## Conflict of interest

The authors declare that the research was conducted in the absence of any commercial or financial relationships that could be construed as a potential conflict of interest.

## Publisher's note

All claims expressed in this article are solely those of the authors and do not necessarily represent those of their affiliated organizations, or those of the publisher, the editors and the reviewers. Any product that may be evaluated in this article, or claim that may be made by its manufacturer, is not guaranteed or endorsed by the publisher.

## References

[1] Cevhertas L Ogulur I Maurer DJ Burla D Ding M Jansen K. Advances and recent developments in asthma in 2020. Allergy. (2020) 75:3124–46. 10.1111/all.14607 32997808

[2] Seys SF Quirce S Agache I Akdis CA Alvaro-Lozano M Antolín-Amérigo D. Severe asthma: entering an era of new concepts and emerging therapies: highlights of the 4th international severe asthma forum, Madrid, 2018. Allergy. (2019) 74:2244–8. 10.1111/all.13843 31021446

[3] Kaur R Chupp G. Phenotypes and endotypes of adult asthma: moving toward precision medicine. J Allergy Clin Immunol. (2019) 144:1–12.25. 10.1016/j.jaci.2019.05.031 31277742

[4] Wenzel SE. Asthma phenotypes: the evolution from clinical to molecular approaches. Nat Med. (2012) 18:716–25. 10.1038/nm.2678 22561835

[5] Fitzpatrick AM Moore WC. Severe asthma phenotypes - how should they guide evaluation and treatment? J Allergy Clin Immunol Pract. (2017) 5:901–8. 10.1016/j.jaip.2017.05.015 28689840 PMC5541906

[6] de Groot JC Storm H Amelink M de Nijs SB Eichhorn E Reitsma BH. Clinical profile of patients with adult-onset eosinophilic asthma. ERJ Open Res. (2016) 2:00100–2015. 10.1183/23120541.00100-2015 27730197 PMC5005181

[7] Agache I Sugita K Morita H Akdis M Akdis CA. The complex type 2 endotype in allergy and asthma: from laboratory to bedside. Curr Allergy Asthma Rep. (2015) 15:29. 10.1007/s11882-015-0529-x 26141574

[8] Agache I Akdis CA. Precision medicine and phenotypes, endotypes, genotypes, regiotypes, and theratypes of allergic diseases. J Clin Invest. (2019) 129:1493–503. 10.1172/JCI124611 30855278 PMC6436902

[9] Chung KF Adcock IM. Precision medicine for the discovery of treatable mechanisms in severe asthma. Allergy. (2019) 74:1649–59. 10.1111/all.13771 30865306

[10] Breiteneder H Peng YQ Agache I Diamant Z Eiwegger T Fokkens WJ. Biomarkers for diagnosis and prediction of therapy responses in allergic diseases and asthma. Allergy. (2020) 75:3039–68. 10.1111/all.14582 32893900 PMC7756301

[11] Agache I Rogozea L. Asthma biomarkers: do they bring precision medicine closer to the clinic? Allergy Asthma Immunol Res. (2017) 9:466–76. 10.4168/aair.2017.9.6.466 28913985 PMC5603474

[12] Papi A Brightling C Pedersen SE Reddel HK. Asthma. Lancet. (2018) 391:783–800. 10.1016/S0140-6736(17)33311-1 29273246

[13] Castillo JR Peters SP Busse WW. Asthma exacerbations: pathogenesis, prevention, and treatment. J Allergy Clin Immunol Pract. (2017) 5:918–27. 10.1016/j.jaip.2017.05.001 28689842 PMC5950727

[14] Bousquet J Rabe K Humbert M Chung KF Berger W Fox H et al. Predicting and evaluating response to omalizumab in patients with severe allergic asthma. Respir Med. (2007) 101:1483–92. 10.1016/j.rmed.2007.01.011 17339107

[15] Ortega HG Liu MC Pavord ID Brusselle GG FitzGerald JM Chetta A. Mepolizumab treatment in patients with severe eosinophilic asthma. N Engl J Med. (2014) 371:1198–207. 10.1056/NEJMoa1403290 25199059

[16] FitzGerald JM Bleecker ER Menzies-Gow A Zangrilli JG Hirsch I Metcalfe P. Predictors of enhanced response with benralizumab for patients with severe asthma: pooled analysis of the SIROCCO and CALIMA studies. Lancet Respir Med. (2018) 6:51–64. 10.1016/S2213-2600(17)30344-2 28919200

[17] Castro M Corren J Pavord ID Maspero J Wenzel S Rabe KF. Dupilumab efficacy and safety in moderate-to-severe uncontrolled asthma. N Engl J Med. (2018) 378:2486–96. 10.1056/NEJMoa1804092 29782217

[18] McDowell PJ Heaney LG. Different endotypes and phenotypes drive the heterogeneity in severe asthma. Allergy. (2020) 75:302–10. 10.1111/all.13966 31267562

[19] Kuruvilla ME Lee FE-H Lee GB. Understanding asthma phenotypes, endotypes, and mechanisms of disease. Clin Rev Allergy Immunol. (2019) 56:219–33. 10.1007/s12016-018-8712-1 30206782 PMC6411459

[20] Pavlidis S Takahashi K Ng Kee Kwong F Xie J Hoda U Sun K. “T2-high” in severe asthma related to blood eosinophil, exhaled nitric oxide and serum periostin. Eur Respir J. (2019) 53:1800938. 10.1183/13993003.00938-2018 30578390

[21] Rupani H Fong WCG Kyyaly A Kurukulaaratchy RJ. Recent Insights into the management of inflammation in asthma. J Inflamm Res. (2021) 14:4371–97. 10.2147/JIR.S295038 34511973 PMC8421249

[22] Chung KF. Airway smooth muscle cells: contributing to and regulating airway mucosal inflammation? Eur Respir J. (2000) 15:961–8. 10.1034/j.1399-3003.2000.15e26.x 10853867

[23] Pelaia C Paoletti G Puggioni F Racca F Pelaia G Canonica GW. Interleukin-5 in the pathophysiology of severe asthma. Front Physiol. (2019) 10:1514. 10.3389/fphys.2019.01514 31920718 PMC6927944

[24] Louis R Sele J Henket M Cataldo D Bettiol J Seiden L. Sputum eosinophil count in a large population of patients with mild to moderate steroid-naïve asthma: distribution and relationship with methacholine bronchial hyperresponsiveness. Allergy. (2002) 57:907–12. 10.1034/j.1398-9995.2002.23608.x 12269936

[25] Schleich FN Chevremont A Paulus V Henket M Manise M Seidel L. Importance of concomitant local and systemic eosinophilia in uncontrolled asthma. Eur Respir J. (2014) 44:97–108. 10.1183/09031936.00201813 24525441

[26] Rhyou HI Nam YH. Predictive factors of response to inhaled corticosteroids in newly diagnosed asthma: a real-world observational study. Ann Allergy Asthma Immunol. (2020) 125:177–81. 10.1016/j.anai.2020.04.025 32371244

[27] Green RH Brightling CE McKenna S Hargadon B Parker D Bradding P. Asthma exacerbations and sputum eosinophil counts: a randomised controlled trial. Lancet. (2002) 360:1715–21. 10.1016/S0140-6736(02)11679-5 12480423

[28] Jayaram L Pizzichini MM Cook RJ Boulet LP Lemière C Pizzichini E. Determining asthma treatment by monitoring sputum cell counts: effect on exacerbations. Eur Respir J. (2006) 27:483–94. 10.1183/09031936.06.00137704 16507847

[29] Pizzichini M Efthimiadis A Dolovich J Hargreave FE. Measuring airway inflammation in asthma: eosinophils and eosinophilic cationic protein in induced sputum compared with peripheral blood. J Allergy Clin Immunol. (1997) 99:539–44. 10.1016/S0091-6749(97)70082-4 9111500

[30] Hastie AT Mauger DT Denlinger LC Coverstone A Castro M Erzurum S. Mixed sputum granulocyte longitudinal impact on lung function in the severe asthma research program. Am J Respir Crit Care Med. (2021) 203:882–92. 10.1164/rccm.202009-3713OC 33545021 PMC8017570

[31] Pavord ID Korn S Howarth P Bleecker ER Buhl R Keene ON. Mepolizumab for severe eosinophilic asthma (DREAM): a multicentre, double-blind, placebo-controlled trial. Lancet. (2012) 380:651–9. 10.1016/S0140-6736(12)60988-X 22901886

[32] Pavord ID Buhl R Kraft M Prazma CM Price RG Howarth PH. Evaluation of sputum eosinophil count as a predictor of treatment response to mepolizumab. ERJ Open Res. (2022) 8:00560–2021. 10.1183/23120541.00560-2021 35509441 PMC9062111

[33] Wagener AH de Nijs SB Lutter R Sousa AR Weersink EJ Bel EH. External validation of blood eosinophils, FE(NO) and serum periostin as surrogates for sputum eosinophils in asthma. Thorax. (2015) 70:115–20. 10.1136/thoraxjnl-2014-205634 25422384

[34] Price DB Rigazio A Campbell JD Bleecker ER Corrigan C Thomas M. Blood eosinophil count and prospective annual asthma disease burden: a UK cohort study. Lancet Respir Med. (2015) 3:849–58. 10.1016/S2213-2600(15)00367-7 26493938

[35] Katz LE Gleich GJ Hartley BF Yancey SW Ortega HG. Blood eosinophil count is a useful biomarker to identify patients with severe eosinophilic asthma. Ann Am Thorac Soc. (2014) 11:531–6. 10.1513/AnnalsATS.201310-354OC 24606022

[36] Brusselle G Nicolini G Santoro L Guastalla D Papi A. BDP/formoterol MART asthma exacerbation benefit increases with blood eosinophil level. Eur Respir J. (2021) 58:2004098. 10.1183/13993003.040982020 33737409

[37] Ortega HG Yancey SW Mayer B Gunsoy NB Keene O Bleecker ER. Severe eosinophilic asthma treated with mepolizumab stratified by baseline eosinophil thresholds: a secondary analysis of the DREAM and MENSA studies. Lancet Respir Med. (2016) 4:549–56. 10.1016/S2213-2600(16)30031-5 27177493

[38] Castro M Zangrilli J Wechsler ME Bateman ED Brusselle G Bardin P. Reslizumab for inadequately controlled asthma with elevated blood eosinophil counts: results from two multicentre, parallel, double-blind, randomised, placebo-controlled, phase 3 trials. Lancet Respir Med. (2015) 3:355–66. 10.1016/S2213-2600(15)00042-9 25736990

[39] Bjermer L Lemiere C Maspero J Weiss S Zangrilli J Germinaro M. Reslizumab for inadequately controlled asthma with elevated blood eosinophil levels: a randomized phase 3 study. Chest. (2016) 150:789–98. 10.1016/j.chest.2016.03.032 27056586

[40] Yancey SW Keene ON Albers FC Ortega H Bates S Bleecker ER. Biomarkers for severe eosinophilic asthma. J Allergy Clin Immunol. (2017) 140:1509–18. 10.1016/j.jaci.2017.10.005 29221581

[41] Hastie AT Moore WC Li H Rector BM Ortega VE Pascual RM. Biomarker surrogates do not accurately predict sputum eosinophil and neutrophil percentages in asthmatic subjects. J Allergy Clin. Immunol. (2013) 132:72–80.e12 10.1016/j.jaci.2013.03.044 23706399 PMC3704048

[42] Chipps BE Jarjour N Calhoun WJ Iqbal A Haselkorn T Yang M. A comprehensive analysis of the stability of blood eosinophil levels. Ann Am Thorac Soc. (2021) 18:1978–87. 10.1513/AnnalsATS.202010-1249OC 33891831 PMC8641810

[43] Wechsler ME Munitz A Ackerman SJ Drake MG Jackson DJ Wardlaw AJ. Eosinophils in health and disease: a state-of-the-art review. Mayo Clin Proc. (2021) 96:2694–707. 10.1016/j.mayocp.2021.04.025 34538424

[44] Ricciardolo FL. Revisiting the role of exhaled nitric oxide in asthma. Curr Opin Pulm Med. (2014) 20:53–9. 10.1097/MCP.0000000000000006 24275926

[45] Mattes J Storm van's Gravesande K Reining U Alving K Ihorst G Henschen M. NO in exhaled air is correlated with markers of eosinophilic airway inflammation in corticosteroid-dependent childhood asthma. Eur Respir J. (1999) 13:1391–5. 10.1183/09031936.99.13613969 10445617

[46] Cloutier MM Baptist AP Blake KV Brooks EG Bryant-Stephens T DiMango E. 2020 focused updates to the asthma management guidelines: a report from the National Asthma Education and Prevention Program Coordinating Committee Expert Panel Working Group. J Allergy Clin Immunol. (2020) 146:1217–70. 10.1016/j.jaci.2020.10.003 33280709 PMC7924476

[47] Dweik RA Boggs PB Erzurum SC Irvin CG Leigh MW Lundberg JO. An official ATS clinical practice guideline: interpretation of exhaled nitric oxide levels (FeNO) for clinical applications. Am J Respir Crit Care Med. (2011) 184:602–15. 10.1164/rccm.9120-11ST 21885636 PMC4408724

[48] Busse WW Wenzel SE Casale TB FitzGerald JM Rice MS Daizadeh N. Baseline FeNO as a prognostic biomarker for subsequent severe asthma exacerbations in patients with uncontrolled, moderate-to-severe asthma receiving placebo in the LIBERTY ASTHMA QUEST study: a post-hoc analysis. Lancet Respir Med. (2021) 9:1165–73. 10.1016/S2213-2600(21)00124-7 34181876

[49] Coumou H Westerhof GA de Nijs SB Zwinderman AH Bel EH. Predictors of accelerated decline in lung function in adult-onset asthma. Eur Respir J. (2018) 51:1701785. 10.1183/13993003.01785-2017 29444915

[50] Sverrild A Porsbjerg C Thomsen SF Backer V. Airway hyperresponsive-ness to mannitol and methacholine and exhaled nitric oxide: a random-sample population study. J Allergy Clin Immunol. (2010) 126:952–8. 10.1016/j.jaci.2010.08.028 20934208

[51] McNicholl DM Stevenson M McGarvey LP Heaney LG. The utility of fractional exhaled nitric oxide suppression in the identification of nonadherence in difficult asthma. Am J Respir Crit Care Med. (2012) 186:1102–8. 10.1164/rccm.201204-0587OC 23024023

[52] van Rensen EL Straathof KC Veselic-Charvat MA Zwinderman AH Bel EH Sterk PJ. Effect of inhaled steroids on airway hyperresponsiveness, sputum eosinophils, and exhaled nitric oxide levels in patients with asthma. Thorax. (1999) 54:403–8. 10.1136/thx.54.5.403 10212103 PMC1763792

[53] Powell H Murphy VE Taylor DR Hensley MJ McCaffery K Giles W. Management of asthma in pregnancy guided by measurement of fraction of exhaled nitric oxide: a double-blind, randomised controlled trial. Lancet. (2011) 378:983–90. 10.1016/S0140-6736(11)60971-9 21907861

[54] Bernholm KF Homøe AS Meteran H Jensen CB Porsbjerg C Backer V. FeNO-based asthma management results in faster improvement of airway hyperresponsiveness. ERJ Open Res. (2018) 4:00147-2017. 10.1183/23120541.00147-2017 30302333 PMC6168761

[55] Price DB Buhl R Chan A Freeman D Gardener E Godley C. Fractional exhaled nitric oxide as a predictor of response to inhaled corticosteroids in patients with non-specific respiratory symptoms and insignificant bronchodilator reversibility: a randomised controlled trial. Lancet Respir Med. (2018) 6:29–39. 10.1016/S2213-2600(17)30424-1 29108938

[56] Jeppegaard M Veidal S Sverrild A Backer V Porsbjerg C. Validation of ATS clinical practice guideline cut-points for FeNO in asthma. Respir Med. (2018) 144:22–9. 10.1016/j.rmed.2018.09.014 30366580

[57] Sharma S Kathuria PC Gupta CK Nordling K Ghosh B Singh AB. Total serum immunoglobulin E levels in a case–control study in asthmatic/allergic patients, their family members, and healthy subjects from India. Clin Exp Allergy. (2006) 36:1019–27. 10.1111/j.1365-2222.2006.02525.x 16911358

[58] Busse W Corren J Lanier BQ McAlary M Fowler-Taylor A Cioppa GD. Omalizumab, anti-IgE recombinant humanized monoclonal antibody, for the treatment of severe allergic asthma. J Allergy Clin Immunol. (2001) 108:184–90. 10.1067/mai.2001.117880 11496232

[59] Chu SY Horton HM Pong E Leung IW Chen H Nguyen DH. Reduction of total IgE by targeted coengagement of IgE B-cell receptor and FcγRIIb with Fc-engineered antibody. J Allergy Clin Immunol. (2012) 129:1102–15. 10.1016/j.jaci.2011.11.029 22257644

[60] de Marco R Marcon A Jarvis D Accordini S Bugiani M Cazzoletti L. Inhaled steroids are associated with reduced lung function decline in subjects with asthma with elevated total IgE. J Allergy Clin Immunol. (2007) 119:611–7. 10.1016/j.jaci.2006.11.696 17258304

[61] Karmaus W Mukherjee N Janjanam VD Chen S Zhang H Roberts G. Distinctive lung function trajectories from age 10 to 26 years in men and women and associated early life risk factors - a birth cohort study. Respir Res. (2019) 20:98. 10.1186/s12931-019-1068-0 31118050 PMC6532227

[62] Belgrave DCM Granell R Turner SW Curtin JA Buchan IE Le Souëf PN. Lung function trajectories from pre-school age to adulthood and their associations with early life factors: a retrospective analysis of three population-based birth cohort studies. Lancet Respir Med. (2018) 6:526–34. 10.1016/S2213-2600(18)30099-7 29628377

[63] McGeachie MJ Yates KP Zhou X Guo F Sternberg AL Van Natta ML. Patterns of growth and decline in lung function in persistent childhood asthma. N Engl J Med. (2016) 374:1842–52. 10.1056/NEJMoa1513737 27168434 PMC5032024

[64] Fitzpatrick AM Jackson DJ Mauger DT Boehmer SJ Phipatanakul W Sheehan WJ. NIH/NHLBI AsthmaNet. Individualized therapy for persistent asthma in young children. J Allergy Clin Immunol. (2016) 138:1608–18.e12. 10.1016/j.jaci.2016.09.028 27777180 PMC5148729

[65] Liu AH Anderson WC III Dutmer CM Searing DA Szefler SJ. Advances in asthma 2015: across the lifespan. J Allergy Clin Immunol. (2016) 138:397–404. 10.1016/j.jaci.2016.06.013 27497278

[66] Wang E Wechsler ME Tran TN Heaney LG Jones RC Menzies-Gow AN. Characterization of severe asthma worldwide: data from the international severe asthma registry. Chest. (2020) 157:790–804. 10.1016/j.chest.2019.10.053 31785254

[67] Borish L Chipps B Deniz Y Gujrathi S Zheng B Dolan CM TENOR Study Group. Total serum IgE levels in a large cohort of patients with severe or difficult-to-treat asthma. Ann Allergy Asthma Immunol. (2005) 95:247–53. 10.1016/S1081-1206(10)61221-5 16200815

[68] Hanania NA Wenzel S Rosén K Hsieh HJ Mosesova S Choy DF. Exploring the effects of omalizumab in allergic asthma: an analysis of biomarkers in the EXTRA study. Am J Respir Crit Care Med. (2013) 187:804–11. 10.1164/rccm.201208-1414OC 23471469

[69] Maselli D Diaz J Peters J. Omalizumab in asthmatics with IgE levels > 700 IU/mL. Eur Respir J. (2011) 38:266. Available online at: https://erj.ersjournals.com/content/38/Suppl_55/p266

[70] Kocks JWH Andringa HJH van Heijst E Louis R Ojanguren Arranz I Riemersma RA. Aeroallergen sensitization for detecting asthma in primary care: a diagnostic test accuracy study. Clin Exp Allergy. (2021) 51:1080–4. 10.1111/cea.13888 33914988 PMC8453944

[71] Matsui EC Sampson HA Bahnson HT Gruchalla RS Pongracic JA Teach SJ. Allergen-specific IgE as a biomarker of exposure plus sensitization in inner-city adolescents with asthma. Allergy. (2010) 65:1414–22. 10.1111/j.1398-9995.2010.02412.x 20560910 PMC3345161

[72] Zoratti EM Krouse RZ Babineau DC Pongracic JA O'Connor GT Wood RA. Asthma phenotypes in inner-city children. J Allergy Clin Immunol. (2016) 138:1016–29. 10.1016/j.jaci.2016.06.061 27720016 PMC5104222

[73] Izuhara K Nunomura S Nanri Y Ono J Takai M Kawaguchi A. Periostin: an emerging biomarker for allergic diseases. Allergy. (2019)74:2116–28. 10.1111/all.13814 30964557

[74] Takayama G Arima K Kanaji T Toda S Tanaka H Shoji S. Periostin: a novel component of subepithelial fibrosis of bronchial asthma downstream of IL-4 and IL-13 signals. J Allergy Clin Immunol. (2006) 118:98–104. 10.1016/j.jaci.2006.02.046 16815144

[75] Takahashi K Meguro K Kawashima H Kashiwakuma D Kagami SI Ohta S. Serum periostin levels serve as a biomarker for both eosinophilic airway inflammation and fixed airflow limitation in well-controlled asthmatics. J Asthma. (2018) 56:236–43. 10.1080/02770903.2018.1455855 29648484

[76] Mansur AH Srivastava S Sahal A. Disconnect of type 2 biomarkers in severe asthma; dominated by FeNO as a predictor of exacerbations and periostin as predictor of reduced lung function. Respir Med. (2018) 143:31–8. 10.1016/j.rmed.2018.08.005 30261989

[77] Hoshino M Ohtawa J Akitsu K. Effect of treatment with inhaled corticosteroid on serum periostin levels in asthma. Respirology. (2015) 21:297–303. 10.1111/resp.12687 26607392

[78] Jia G Erickson RW Choy DF Mosesova S Wu LC Solberg OD et al. Periostin is a systemic biomarker of eosinophilic airway inflammation in asthmatic patients. J Allergy Clin Immunol. (2012) 130:647–654.e610. 10.1016/j.jaci.2012.06.025 22857879 PMC3626285

[79] Caswell-Smith R Hosking A Cripps T Holweg C Matthews J Holliday M. Periostin Study Team. Reference ranges for serum periostin in a population without asthma or chronic obstructive pulmonary disease. Clin Exp Allergy. (2016) 46:1303–14. 10.1111/cea.12763 27237923

[80] Corren J Lemanske RF Hanania NA Korenblat PE Parsey MV Arron J. Lebrikizumab treatment in adults with asthma. N Engl J Med. (2011) 365:1088–98. 10.1056/NEJMoa1106469 21812663

[81] Brightling C Chanez P Leigh R O'Byrne P Korn S She D. Efficacy and safety of tralokinumab in patients with severe uncontrolled asthma: A randomised, double-blind, placebo-controlled, phase 2b trial. Lancet Respir Med. (2015) 3:692–701. 10.1016/S2213-2600(15)00197-6 26231288

[82] Fitzpatrick AM Chipps BE Holguin F Woodruff PG. T2-”low” asthma: overview and management strategies. J Allergy Clin Immunol Pract. (2020) 8:452–63. 10.1016/j.jaip.2019.11.006 32037109

[83] Jatakanon A Uasuf C Maziak W Lim S Chung KF Barnes PJ. Neutrophilic inflammation in severe persistent asthma. Am J Respir Crit Care Med. (1999) 160:1532–9. 10.1164/ajrccm.160.5.9806170 10556116

[84] Gibson PG Foster PS. Neutrophilic asthma: Welcome back! Eur Respir J. (2019) 54:1901846. 10.1183/13993003.01846-2019 31699782

[85] Belda J Leigh R Parameswaran K O'Byrne PM Sears MR Hargreave FE. Induced Sputum Cell Counts in Healthy Adults. Am J Respir Crit Care Med. (2000) 161:475–8. 10.1164/ajrccm.161.2.9903097 10673188

[86] Brooks CR Gibson P Douwes J Van Dalen CJ Simpson JL. Relationship between airway neutrophilia and ageing in asthmatics and non-asthmatics. Respirology. (2013) 18:857–65. 10.1111/resp.12079 23490307

[87] Sze E Bhalla A Nair P. Mechanisms and therapeutic strategies for non-T2 asthma. Allergy. (2020) 75:311–25. 10.1111/all.13985 31309578

[88] Taylor S Leong LEX Choo JM Wesselingh S Yang I Upham J. Inflammatory phenotypes in patients with severe asthma are associated with distinct airway microbiology. J Allergy Clin Immunol. (2018) 141:94–103.e15 10.1016/j.jaci.2017.03.044 28479329

[89] Agache I Ciobanu C Agache C Anghel M. Increased serum IL-17 is an independent risk factor for severe asthma. Respir Med. (2010) 104:1131–7. 10.1016/j.rmed.2010.02.018 20338742

[90] Fu JJ Baines KJ Wood L Gibson P. Systemic inflammation is associated with differential gene expression and airway neutrophilia in asthma. OMICS. (2013) 17:187–99. 10.1089/omi.2012.0104 23438328

[91] Tliba O Panettieri RA. Paucigranulocytic asthma: uncoupling of airway obstruction from inflammation. J Allergy Clin Immunol. (2019) 143:1287–94. 10.1016/j.jaci.2018.06.008 29928921 PMC6301131

[92] Papaioannou AI Fouka E Ntontsi P Stratakos G Papiris S. Paucigranulocytic asthma: potential pathogenetic mechanisms, clinical features and therapeutic management. J Pers Med. (2022) 12:850. 10.3390/jpm12050850 35629272 PMC9145917

[93] Marshall CL Hasani K Mookherjee N. Immunobiology of steroid-unresponsive severe asthma. Front Allergy. (2021) 2:718267. 10.3389/falgy.2021.718267 35387021 PMC8974815

[94] Berry M Morgan A Shaw D Parker D Green R Brightling C. Pathological features and inhaled corticosteroid response of eosinophilic and non-eosinophilic asthma. Thorax. (2007) 62:1043–9. 10.1136/thx.2006.073429 17356056 PMC2094295

[95] Demarche S Schleich F Henket M Paulus V Louis R Van Hees T. Step-down of inhaled corticosteroids in non-eosinophilic asthma: A prospective trial in real life. Clin Exp Allergy. (2018) 48:525–35. 10.1111/cea.13106 29383782

[96] Agusti A Bafadhel M Beasley R. Precision medicine in airway diseases: moving to clinical practice. Eur Respir J. (2017) 50:1701655. 10.1183/13993003.01655-2017 29051276

[97] Chaudhuri R Livingston E McMahon AD Lafferty J Fraser I Spears M. Effects of smoking cessation on lung function and airway inflammation in smokers with asthma. Am J Respir Crit Care Med. (2006) 174:127–33. 10.1164/rccm.200510-1589OC 16645173

[98] Dias-Júnior SA Reis M de Carvalho-Pinto RM Stelmach R Halpern A Cukier A. Effects of weight loss on asthma control in obese patients with severe asthma. Eur Respir J. (2014) 43:1368–77. 10.1183/09031936.00053413 24232701

[99] Gibson PG Yang I Upham J Reynolds PN Hodge S James AL. Effect of azithromycin on asthma exacerbations and quality of life in adults with persistent uncontrolled asthma (AMAZES): A randomised, double-blind, placebo-controlled trial. Lancet. (2017) 390:659–68. 10.1016/S0140-6736(17)31281-3 28687413

[100] Esteban-Gorgojo I Antolín-Amérigo D Domínguez-Ortega J Quirce S. Non-eosinophilic asthma: current perspectives. J Asthma Allergy. (2018) 11:267–81. 10.2147/JAA.S153097 30464537 PMC6211579

[101] Ivanova O Richards LB Vijverberg SJ Neerincx AH Sinha A Sterk PJ. What did we learn from multiple omics studies in asthma? Allergy. (2019) 74:2129–45. 10.1111/all.13833 31004501

[102] Hur GY Pham A Miller M Weng N Hu J Kurten RC. ORMDL3 but not neighboring 17q21 gene LRRC3C is expressed in human lungs and lung cells of asthmatics. Allergy. (2020) 75:2061–5. 10.1111/all.14243 32086831 PMC7387186

[103] Lemonnier N Melén E Jiang Y Joly S Ménard C Aguilar D. A novel whole blood gene expression signature for asthma, dermatitis, and rhinitis multimorbidity in children and adolescents. Allergy. (2020) 75:3248–60. 10.1111/all.14314 32277847 PMC9302020

[104] Dijk FN Vijverberg SJ Hernandez-Pacheco N Repnik K Karimi L Mitratza M. IL1RL1 gene variations are associated with asthma exacerbations in children and adolescents using inhaled corticosteroids. Allergy. (2020) 75:984–9. 10.1111/all.14125 31755552 PMC7176513

[105] Diamant Z Vijverberg S Alving K Bakirtas A Bjermer L Custovic A. Toward clinically applicable biomarkers for asthma: an EAACI position paper. Allergy. (2019) 74:1835–51. 10.1111/all.13806 30953574

[106] Zounemat Kermani N Saqi M Agapow P Pavlidis S Kuo C Tan KS. U- BIOPRED Project Team. Type 2- low asthma phenotypes by integration of sputum transcriptomics and serum proteomics. Allergy. (2021) 76:380–3. 10.1111/all.14573 32865817

[107] Phillips M Gleeson K Hughes JM Greenberg J Cataneo RN Baker L. Volatile organic compounds in breath as markers of lung cancer: a cross-sectional study. Lancet. (1999) 353:1930–3. 10.1016/S0140-6736(98)07552-7 10371572

[108] Azim A Barber C Dennison P Riley J Howarth P. Exhaled volatile organic compounds in adult asthma: a systematic review. Eur Respir J. (2019) 54:1900056. 10.1183/13993003.00056-2019 31273044

[109] Horváth I Barnes PJ Loukides S Sterk PJ Högman M Olin AC. A European Respiratory Society technical standard: exhaled biomarkers in lung disease. Eur Respir J. (2017) 49:1600965. 10.1183/13993003.00965-2016 28446552

[110] Ibrahim W Carr L Cordell R Wilde MJ Salman D Monks PS. Breathomics for the clinician: the use of volatile organic compounds in respiratory diseases. Thorax. (2021) 76:514–21. 10.1136/thoraxjnl-2020-215667 33414240 PMC7611078

[111] Brinkman P van de Pol MA Gerritsen MG Bos LD Dekker T Smids BS et al. Exhaled breath profiles in the monitoring of loss of control and clinical recovery in asthma. Clin Exp Allergy. (2017) 47:1159–69. 10.1111/cea.12965 28626990

[112] Peel AM Wilkinson M Sinha A Loke YK Fowler SJ Wilson AM. Volatile organic compounds associated with diagnosis and disease characteristics in asthma - A systematic review. Respir Med. (2020) 169:105984. 10.1016/j.rmed.2020.105984 32510334

[113] Abdel-Aziz MI de Vries R Lammers A Xu B Neerincx AH Vijverberg SJH. Cross- sectional biomarker comparisons in asthma monitoring using a longitudinal design: the eNose premise. Allergy. (2020) 75:2690–3. 10.1111/all.14354 32542855

[114] Nieto-Fontarigo JJ González-Barcala FJ Andrade-Bulos LJ San-José ME Cruz MJ Valdés-Cuadrado L. iTRAQ- based proteomic analysis reveals potential serum biomarkers of allergic and nonallergic asthma. Allergy. (2020) 75:3171–83. 10.1111/all.14406 32424932

[115] Tseliou E Bessa V Hillas G Delimpoura V Papadaki G Roussos C. Exhaled nitric oxide and exhaled breath condensate pH in severe refractory asthma. Chest. (2010) 138:107–13. 10.1378/chest.09-1257 20173051

[116] Hillas G Kostikas K Mantzouranis K Bessa V Kontogianni K Papadaki G. Exhaled nitric oxide and exhaled breath condensate pH as predictors of sputum cell counts in optimally treated asthmatic smokers. Respirology. (2011) 16:811–8. 10.1111/j.1440-1843.2011.01984.x 21545371

[117] Liu L Teague WG Erzurum S Fitzpatrick A Mantri S Dweik RA. National Heart, Lung, and Blood Institute Severe Asthma Research Program (SARP). Determinants of exhaled breath condensate pH in a large population with asthma. Chest. (2011) 139:328–36. 10.1378/chest.10-0163 20966042 PMC3032364

[118] Holz O Waschki B Watz H Kirsten A Abdo M Pedersen F. Breath volatile organic compounds and inflammatory markers in adult asthma patients: negative results from the ALLIANCE cohort. Eur Respir J. (2021) 57:2002127. 10.1183/13993003.02127-2020 33008938 PMC7876421

[119] Walter J O'Mahony L. The importance of social networks- An ecological and evolutionary framework to explain the role of microbes in the aetiology of allergy and asthma. Allergy. (2019) 74:2248–51. 10.1111/all.13845 31034612

[120] Sbihi H Boutin RC Cutler C Suen M Finlay BB Turvey SE. Thinking bigger: how early-life environmental exposures shape the gut microbiome and influence the development of asthma and allergic disease. Allergy. (2019) 74:2103–15. 10.1111/all.13812 30964945

[121] Barcik W Pugin B Westermann P Perez NR Ferstl R Wawrzyniak M. Histamine-secreting microbes are increased in the gut of adult asthma patients. J Allergy Clin Immunol. (2016) 138:1491–4.e7. 10.1016/j.jaci.2016.05.049 27576125

[122] Barcik W Pugin B Brescó MS Westermann P Rinaldi A Groeger D. Bacterial secretion of histamine within the gut influences immune responses within the lung. Allergy. (2019) 74:899–909. 10.1111/all.13709 30589936

[123] Azad MB Coneys JG Kozyrskyj AL Field CJ Ramsey CD Becker AB. Probiotic supplementation during pregnancy or infancy for the prevention of asthma and wheeze: systematic review and meta-analysis. BMJ. (2013) 347:f6471. 10.1136/bmj.f6471 24304677 PMC3898421

[124] Wypych TP Marsland BJ. Antibiotics as instigators of microbial dysbiosis: implications for asthma and allergy. Trends Immunol. (2018) 39:697–711. 10.1016/j.it.2018.02.008 29655522

[125] Jensen-Jarolim E Bax HJ Bianchini R Crescioli S Daniels-Wells TR Dombrowicz D. AllergoOncology: opposite outcomes of immune tolerance in allergy and cancer. Allergy. (2018) 73:328–40. 10.1111/all.13311 28921585 PMC6038916

[126] Zhou Y Jackson D Bacharier LB Mauger D Boushey H Castro M. The upper-airway microbiota and loss of asthma control among asthmatic children. Nat Commun. (2019) 10:5714. 10.1038/s41467-019-13698-x 31844063 PMC6915697

[127] Fujimura KE Sitarik AR Havstad S Lin DL Levan S Fadrosh D et al. Neonatal gut microbiota associates with childhood multisensitized atopy and T cell differentiation. Nat Med. (2016) 22:1187–91. 10.1038/nm.4176 27618652 PMC5053876

[128] Huang YJ Nariya S Harris JM Lynch SV Choy DF Arron JR. The airway microbiome in patients with severe asthma: associations with disease features and severity. J Allergy Clin Immunol. (2015) 136:874–84. 10.1016/j.jaci.2015.05.044 26220531 PMC4600429

[129] Lewis G Wang B Shafiei Jahani P Hurrell BP Banie H Aleman Muench GR. Dietary fiber-induced microbial short chain fatty acids suppress ILC2-dependent airway inflammation. Front Immunol. (2019) 10:2051. 10.3389/fimmu.2019.02051 31620118 PMC6760365

[130] Chiu CY Chou HC Chang LC Fan WL Dinh MCV Kuo YL. Integration of metagenomics-metabolomics reveals specific signatures and functions of airway microbiota in mite-sensitized childhood asthma. Allergy. (2020) 75:2846–57. 10.1111/all.14438 32506557

[131] Fricker M McDonald VM Winter NA Baines KJ Wark PAB Simpson JL. Molecular markers of type 2 airway inflammation are similar between eosinophilic severe asthma and eosinophilic COPD. Allergy. (2021) 76:2079–89. 10.1111/all.14741 33470427

[132] Prazma CM Bel EH Price RG Bradford ES Albers FC Yancey SW. Oral corticosteroid dose changes and impact on peripheral blood eosinophil counts in patients with severe eosinophilic asthma: a post hoc analysis. Respir Res. (2019) 20:83. 10.1186/s12931-019-1056-4 31053134 PMC6499981

[133] Durrington HJ Gioan-Tavernier GO Maidstone RJ Krakowiak K Loudon ASI Blaikley JF. Time of day affects eosinophil biomarkers in asthma:implications for diagnosis and treatment. Am J Respir Crit Care Med. (2018) 198:1578–81. 10.1164/rccm.201807-1289LE 30156881 PMC6298638

[134] Korevaar DA Westerhof GA Wang J Cohen JF Spijker R Sterk PJ. Diagnostic accuracy of minimally invasive markers for detection of airway eosinophilia in asthma: a systematic review and meta-analysis. Lancet Respir Med. (2015) 3:290–300. 10.1016/S2213-2600(15)00050-8 25801413

[135] Pavord ID Beasley R Agusti A Anderson GP Bel E Brusselle G. After asthma: redefining airways diseases. Lancet. (2018) 391:350–400. 10.1016/S0140-6736(17)30879-6 28911920

[136] Heaney LG Perez de Llano L Al-Ahmad M Backer V Busby J Canonica GW. Eosinophilic and noneosinophilic asthma: an expert consensus framework to characterize phenotypes in a global real-life severe asthma cohort. Chest. (2021) 160:814–30. 10.1016/j.chest.2021.04.013 33887242

[137] Azim A Newell C Barber C Harvey M Knight D Freeman A. Clinical evaluation of type 2 disease status in a real-world population of difficult to manage asthma using historic electronic healthcare records of blood eosinophil counts. Clin Exp Allergy. (2021) 51:811–20. 10.1111/cea.13841 33528864

[138] Price DB Bosnic-Anticevich S Pavord ID Roche N Halpin DMG Bjermer L. Association of elevated fractional exhaled nitric oxide concentration and blood eosinophil count with severe asthma exacerbations. Clin Transl Allergy. (2019) 9:41. 10.1186/s13601-019-0282-7 31452870 PMC6702739

[139] Buhl R Humbert M Bjermer L Chanez P Heaney LG Pavord I. Severe eosinophilic asthma: a roadmap to consensus. Eur Respir J. (2017) 49:1700634. 10.1183/13993003.00634-2017 28461308

[140] ISAR Study Group. International Severe Asthma Registry (ISAR): mission statement. Chest. (2020) 157:805–14. 10.1016/j.chest.2019.10.051 31838187

[141] Wu W Bleecker E Moore W Busse WW Castro M Chung KF. Unsupervised phenotyping of Severe Asthma Research Program participants using expanded lung data. J Allergy Clin Immunol. (2014) 133:1280–8. 10.1016/j.jaci.2013.11.042 24589344 PMC4038417

[142] Holguin F Cardet JC Chung KF Diver S Ferreira DS Fitzpatrick A. Management of severe asthma: a European respiratory society/ American thoracic society guideline. Eur Respir J. (2020) 55:1900588. 10.1183/13993003.00588-2019 31558662

[143] Agache I Akdis CA Akdis M Canonica GW Casale T Chivato T. EAACI biologicals guidelines-recommendations for severe asthma. Allergy. (2021) 76:14–44. 10.1111/all.14425 32484954

[144] Global Initiative for Asthma. GINA Guidelines. Global Strategy for Asthma Management and Prevention. (2022). Available online at: htpp://www.ginasthma.org/ (accessed July 1, 2022).

